# Gelatin-Based Metamaterial Hydrogel Films with High Conformality for Ultra-Soft Tissue Monitoring

**DOI:** 10.1007/s40820-023-01225-z

**Published:** 2023-11-29

**Authors:** Yuewei Chen, Yanyan Zhou, Zihe Hu, Weiying Lu, Zhuang Li, Ning Gao, Nian Liu, Yuanrong Li, Jing He, Qing Gao, Zhijian Xie, Jiachun Li, Yong He

**Affiliations:** 1 State Key Laboratory of Fluid Power and Mechatronic Systems, School of Mechanical Engineering, Zhejiang University, Hangzhou, 310027 People’s Republic of China; 2 School of Mechanical Engineering, Guizhou University, Guiyang, 550025 People’s Republic of China; 3 Stomatology Hospital, School of Stomatology, Zhejiang University School of Medicine, Clinical Research Center for Oral Diseases of Zhejiang Province, Key Laboratory of Oral Biomedical Research of Zhejiang Province, Cancer Center of Zhejiang University, Hangzhou, 310006 People’s Republic of China

**Keywords:** Implantable hydrogel-based bioelectronics, Conformality, 3D printing, Metamaterial design

## Abstract

Implantable hydrogel-based bioelectronics (IHB) can precisely monitor human health and diagnose diseases. However, achieving biodegradability, biocompatibility, and high conformality with soft tissues poses significant challenges for IHB. Gelatin is the most suitable candidate for IHB since it is a collagen hydrolysate and a substantial part of the extracellular matrix found naturally in most tissues. This study used 3D printing ultrafine fiber networks with metamaterial design to embed into ultra-low elastic modulus hydrogel to create a novel gelatin-based conductive film (GCF) with mechanical programmability. The regulation of GCF nearly covers soft tissue mechanics, an elastic modulus from 20 to 420 kPa, and a Poisson's ratio from − 0.25 to 0.52. The negative Poisson's ratio promotes conformality with soft tissues to improve the efficiency of biological interfaces. The GCF can monitor heartbeat signals and respiratory rate by determining cardiac deformation due to its high conformability. Notably, the gelatin characteristics of the biodegradable GCF enable the sensor to monitor and support tissue restoration. The GCF metamaterial design offers a unique idea for bioelectronics to develop implantable sensors that integrate monitoring and tissue repair and a customized method for endowing implanted sensors to be highly conformal with soft tissues.

**Supplementary Information:**

The online version contains supplementary material available at 10.1007/s40820-023-01225-z.

## Highlights

- Novel customized metamaterial gelatin-based conductive film (GCF) was developed to ensure good biocompatibility and biodegradability for implantable bioelectronics integrating monitoring with tissue repair. The metamaterial property demonstrated high conformal with soft tissues to promote the signal-to-noise ratio.
- The GCF revealed elastic modulus regulated from 20 to 420 kPa and Poisson's ratio from − 0.25 to 0.52. It was fabricated by embedding different 3D printing ultrafine fiber networks with metamaterial design into ultra-low modulus Gelatin methacryloyl hydrogel.

**Supplementary Information:**

The online version contains supplementary material available at 10.1007/s40820-023-01225-z.

## Graphical abstract

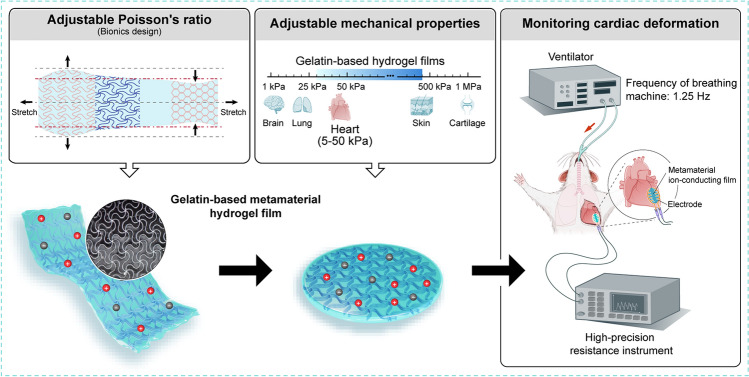

## Introduction

Recently, implantable flexible bioelectronics (IFB) has received substantial attention for monitoring and recording various physiological information of human health or pathological tissues and organs, such as skin [1, 2], brain [3, 4], nerve [5] and heart [6–8]. However, traditional flexible sensor substrates are elastomers [9–12], such as polydimethylsiloxane (PDMS) and Ecoflex. The range of elastomer moduli (1 MPa to 1 GPa) is much more extensive than the modulus of human soft tissues (1 Pa to 1 MPa), leading to inadequate conformality and mechanical mismatch. Mechanical mismatch and poor biocompatibility cause an excessive immune response and impose an imperceptible constraint on routine functions of biological tissues [13]. The severity of mechanical mismatch may damage the tissues. Furthermore, the elastomer's non-degradability makes it unsuitable for implantation. Lee et al. [14] proposed a tensile strain sensor to precisely estimate the strength and direction of strain. Shi et al. [15] developed pressure sensors with outstanding sensitivity and mechanical stability by integrating an ionic gel into the elastomer matrix. However, in their study, the selected elastomer as the substrate was incompatible with the elastic modulus of human soft tissues. For IFB, it is essential to consider tissues' physical characteristics, such as inconsistent geometric features (sizes and surface curvatures) and anisotropy. Moreover, improving the conformality between bioelectronics and tissues provides a stable electronic interface for biological tissues and improves signal accuracy [16]. High conformality is particularly important for monitoring tissues and organs with mechanical deformation and curved surfaces, such as the heart, lungs, and other body parts, as it helps to guarantee effective strain transfer and reduce relative interface motion. However, achieving high conformality between implantable flexible bioelectronics and tissues or organs is challenging.

The stress–strain curves of most biological tissues and organs (such as skin and ligament) have a J-shaped response. Flexible sensors with J-response demonstrate excellent conformality with tissues [17]. Hydrogels have a high-water content, and their mechanical and biological qualities are similar to those of biological tissues. Hydrogel-based bioelectronics offers enhanced compliance and affinity with soft tissues. Therefore, hydrogel-based bioelectronics has attracted growing attention for developing new materials and increasing sensor performance, such as adding conductive nanoparticles to boost conductivity. Li et al. [18] used MXene nanosheets in the PAA–ACC hydrogel polymer network to attain self-healing ability and improve response time.

Notably, biodegradable hydrogel-based bioelectronics reduces the risk of secondary surgery. Nevertheless, as implantable sensors, flexible electronics currently have considerable limitations. The next generation of implantable hydrogel-based bioelectronics (IHB) must be biohydrogel-based bioelectronics, projected to overcome the limitations of intricate mechanical properties and geometric features of human tissues, and a complex biochemical environment in vivo. Biohydrogels have unique advantages over typical elastomers: (1) Biohydrogels are the primary elements of natural extracellular matrix (ECM), offering good biocompatibility, minimal immunogenicity, and non-toxicity. (2) Biohydrogels contain several bioactive motifs, such as matrix metalloproteinase target sequences suitable for cell remodeling, which can be degraded and absorbed by enzymes [19, 20]. Since some motifs can promote cell migration and differentiation, biohydrogels can monitor damaged tissues in real time and promote tissue repair [21, 22]. (3) The exchange of information in vivo differs from standard electrical devices and depends on the electrolyte's ion activity. Biohydrogels have a high-water content and permeability, allowing them to carry medicines, growth factors, and other substances. (4) Biohydrogels have more functional groups, such as hydroxyl, carboxyl, and amino groups, which allow for molecular modification and provide biohydrogels with desired properties.

Gelatin is a collagen hydrolysate that is the major component of natural ECM in most tissues. It contains many biologically active motifs, such as the arginine-glycine-aspartic acid (RGD) sequence that stimulates cell adhesion and a matrix metalloproteinase (MMP) target sequence [23–26]. The current issues associated with IHB may be solved if gelatin can be used as the essential component of IHB. However, many ultra-soft tissues are curvature surfaces with minimal elastic modulus. For instance, the elastic modulus of the heart is 5 ~ 50 kPa [16]. The film made of biohydrogel is highly conformal with soft tissues. Gelatin film has high elasticity and toughness [27], but the elastic modulus of gelatin films is high (≥ 1 MPa) [28–30], which is significantly greater than the elastic modulus of soft tissues. In contrast, the film's toughness is low, and its strength can be reduced. Therefore, it is challenging to manufacture films with high toughness and low elastic modulus. Soft collagen-like fibers are embedded into ultra-low modulus matrices to toughen hydrogels and provide matrix materials with excellent mechanical properties, such as regulating elastic modulus [31, 32], J-shape stress–strain response [33], and anisotropy. Melt Electrowriting (MEW) can produce flexible ultrafine fiber networks (UFNs) with diverse topological structures [33, 34]. The MEW has broad applications in tissue engineering due to controllable fiber deposition and diameter (800 nm–150 µm) [35]. Poly-ɛ-caprolactone (PCL) is an FDA-approved clinical biodegradable material suitable for MEW. Recently, MEW applications have been reported for tissue engineering by the authors of these studies [35–38]. If UFNs with metamaterial design are embedded into the biohydrogel, the biohydrogel with the effect of a negative Poisson's ratio (NPR) may increase the ability of biaxial stretching, minimize stress concentration, and improve conformality with tissues and organs [39].

**Fig. 1:**
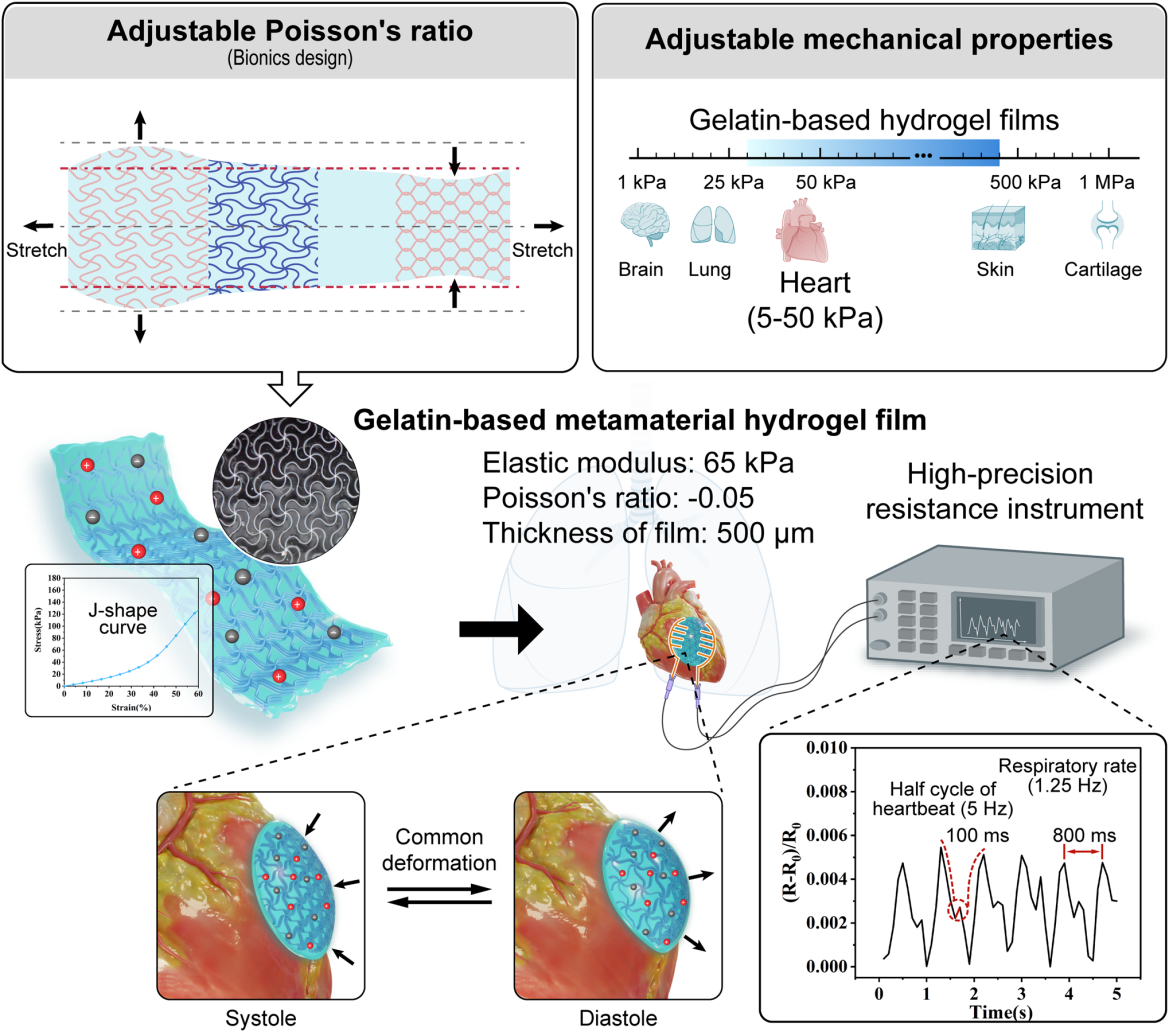
Schematics of a mechanically programmable gelatin-based conductive film for high conformality and its applications as implantable strain sensors to monitor cardiac deformation

Herein, a mechanically programmable gelatin-based conductive film was developed considering bionic design **(**Fig. 1**)**. The design offers an adjustable Poisson's ratio and elastic modulus, which can be conformal with human tissues, especially ultra-soft tissues. In this study, GelMA30 hydrogel was prepared with excellent biocompatibility by controlling the ratio of methacryloyl replacing the amino group on the gelatin molecular chain with 30 percent. The developed hydrogel has an ultra-low modulus, high elasticity, and adhesion as a matrix. To enhance anti-dehydration and conductive function, glycerol and salt ions were added to the hydrogel. Flexible UFNs with metamaterial design printed by MEW were embedded into the GelMA30 matrix to change the elastic modulus of GCF from 20 to 420 kPa and the Poisson's ratio from − 0.25 to 0.52. GCF with cardiac mechanics and NPR was utilized as a flexible strain sensor to monitor the cardiac deformation and acquire data that the mouse's heartbeat and breathing frequency are approximately 5 Hz and 1.25 Hz, respectively. The ability of GCF to facilitate tissue repair was demonstrated through full-thickness skin defect repair.

## Experimental Section

### Materials

GelMA30 was obtained from Suzhou Intelligent Manufacturing Research Institute, Suzhou, China. GelMA30 (10% (w/v)) prepolymer solution was prepared by dissolving freeze-drying GelMA30 in binary solvent containing phenyl-2, 4, 6-trimethylbenzoyl phosphite lithium (0.3% w/v). PCL pellets (CAPA6800, Perstorp Ltd., Sweden) with a molecular weight of 80,000 g mol^−1^ and a melting temperature of 60 °C were the source material for high-resolution 3D printing. Glycerol (purity > 99.9%) with a molecular weight of 92.09 g mol^−1^ was sourced from Sinopharm Chemical Reagent Shanghai Co., Ltd., China.

### Fabrication of Ultrafine Fiber Networks

The UFNs were manufactured by a custom-built MEW printer (EFL-BP6601, Suzhou Intelligent Manufacturing Research Institute, Suzhou, China). PCL pellets were added to a syringe with a 200 μm nozzle. The syringe and nozzle were heated to 120 °C and then maintained for 2 h. The distance between the plate collector and nozzle was 1.8 mm, and the moving speed of the collecting plate was 300 mm min^−1^. Other printing parameters are given in Table 1.

**Table 1:** Printing parameters

Fiber diameter (μm)	Voltage (kV)	Air pressure (kPa)
45	2.87	69
60	3.07	150
80	2.7	350

### Fabrication of Composite Film with UFNs

First, the base film (thickness: 100 μm) was scraped on the glass plate and then pre-crosslinked by light with a wavelength of 405 nm and a power of 90 mW mm^−2^ for 5 s. The middle film (thickness: 300 μm) was scraped on the base film, and UFNs (length × width: 30 mm × 30 mm) were put on the middle film to soak. The middle film was pre-crosslinked for 5 s. Finally, the top film (thickness: 100 μm) was scraped on the middle film. The film was crosslinked for 30 s and then peeled off from the glass plate.

### Images of UFNs and Electrical Measurements

The UFN patterns were scanned by electron microscopy at an acceleration voltage of 3 kV (SEM, Hitachi SU-8010, Japan) after coating UFNs with platinum by a sputter coater (Ion Sputter E-1045, Hitachi, Tokyo, Japan). The fiber diameter was measured by Image J software.

The conductivity of hydrogel was measured by using an electrochemical workstation (CHI760e, China). The ionic conductivity of hydrogel was calculated by the following formula: $\sigma = L/(R \times S)$ (mS/cm). Here, L is the distance between the electrodes, R is the measured resistance, and S is the contact area between the electrode and hydrogel. A universal tensile machine (UTM2203, China) and a high-precision resistance instrument (Keysight, 34465A) were used to test the mechanical and electrical properties of the samples. The test sample size was 20 mm × 15 mm, and the tensile speed of the measuring sensor specification factor was 5 mm min^−1^. In the cyclic test experiment, 500 and 1000 cycles loading and unloading 40% strain at 50 and 100 mm min^−1^, respectively.

### Mechanical Characterization and Adhesive Test

A universal tensile machine (UTM2203, China) was used with a load cell of 20 N. The hydrogel samples were dumbbell-shaped (length: 75 mm, width: 4 mm, thickness: 2 mm), and the tensile speed was 5 mm min^−1^. Gelatin-based films (30 mm × 15 mm × 0.5 mm) were tested at 2 mm min^−1^ tensile speed. The tensile modulus was calculated at a strain of 15%.

A 180° shear peel test was used to analyze the adhesive performance of a hydrogel. The test sample (15 mm × 25 mm × 2 mm) was placed between two substrates, and the substrate was fixed on the surface of the glass sheet. The universal tensile machine (UTM2203, China) was used to pull the glass sheet at a tensile speed of 10 mm min^−1^.

### Characterization of Strain Cloud and Poisson's Ratio

The test sample surfaces (30 mm × 15 mm × 0.5 mm) were painted to form black spots. The samples were stretched by the universal tensile machine (UTM2203, China) at a speed of 2 mm min^−1^, and real-time images were taken with an industrial camera (Zhejiang Dahua Technology Co., Ltd.) at a rate of 1 Hz. DIC software performed strain analysis on the collected images, reconstructed the strain cloud, and calculated the Poisson's ratio using $\upsilon = - E_{XX} /E_{YY}$. E_XX_ is the average Lagrange strain in the axial direction, and E_YY_ is the average Lagrange strain in the tensile direction. The Poisson's ratio of all samples was obtained at 15% strain.

### Characterization of Biocompatibility and Hemolysis

Cell viability was evaluated by Live/Dead and CCK8 assays. The L929 cells were used for the cytocompatibility assay. The cells were seeded at a density of 1 × 10^5^ cells/well on 24 well plates and incubated for 24 h. Then the material was co-cultured with cells via transwell. After culturing for 1, 3, and 5 days, cell proliferation was determined using a Cell Counting Kit-8 (Beyotime) assay (CCK-8) according to the manufacturer's instructions. For Live/Dead, after co-culturing for 1, 3, and 5 days, plates were rinsed twice with PBS and treated with a Live/Dead kit (Bestbio). A fluorescence microscope (Invitrogen, EVOS M5000) was used to observe and photograph cells.

8 mL of rabbit whole blood with sodium citrate(Bestbio, China) was diluted with 0.9% normal saline, then incubated with the material for 30 min at 37 °C. The liquid was removed and centrifuged at 800 g for 5 min; The OD of the supernatant was read at a wavelength of 560 nm. The blood was placed into the deionized water and physiological saline as the positive and negative control.

### Measurement of Cardiac Deformation

The animal experiment process strictly followed the protocols approved by the Institutional Animal Care and Use Committee of Zhejiang province (ZJCLA-IACUC-20030100). For the cardiac deformation, 350 g rats were used. First, 2% pentobarbital was injected into the abdominal cavity of rats to induce anesthesia, and then tracheal intubation was performed on the rats while sevoflurane was used to maintain anesthesia. Next, thoracotomy was performed from the left chest wall of the rats to expose the heart and remove the pericardium. The sensor (diameter 10 mm) was attached to the cardiac surface, and the change in resistance was recorded by a high-precision resistance instrument.

### Wound Healing and Monitoring in vivo

The animal experiments were approved by the Institutional Animal Care and Use Committee of Zhejiang province (ZJCLA-IACUC-20030100). Thirty male rats (8-week-old Sprague–Dawley rats weighing 250–300 g) were used in the experiment. The rats were separated into the Blank and Material groups (UFNs + GelMA30 hydrogel). The rats were anesthetized by intraperitoneal injection of pentobarbital sodium at 50 mg kg^−1^. Circles with 2.0 cm diameter full-thickness skin defects were made on the back of the rat. The materials were sutured on the wound, and the gauze covered the wound to prevent rats from biting. The wounds of the rats in the blank group were covered with gauze. At the same time, initial wound monitoring was performed with a high-precision resistance instrument on days 0, 1, 3, 5 and 7. The recovery of the wounds was evaluated on days 0, 3, 7, and 14, and images were taken with a digital camera. The areas were quantitatively analyzed by Image J software.

The rats were sacrificed by CO_2_ on days 3, 7, and 14 (five rats at each time point). After the operation, skin samples were collected and paraffin-embedded. After sectioning, the sections were stained with hematoxylin–eosin (H & E) and Masson trichrome stains. Immunohistochemical staining of CD31 (1: 2000, 28,083–1-AP, protein tech) was used to analyze angiogenesis. The stained sections were observed by Vs200 (Olympus, Japan) and analyzed by Image J software.

## Results and Discussion

### Physical Properties of GelMA30 Hydrogel

Gelatin methacryloyl (GelMA) hydrogel was obtained by substituting amino and hydroxyl groups on the molecular chains of gelatin with methacryloyl. GelMA exhibits high biocompatibility at any concentration or crosslinking degree, but a high crosslinking degree of GelMA leads to weak cytocompatibility.

**Fig. 2:**
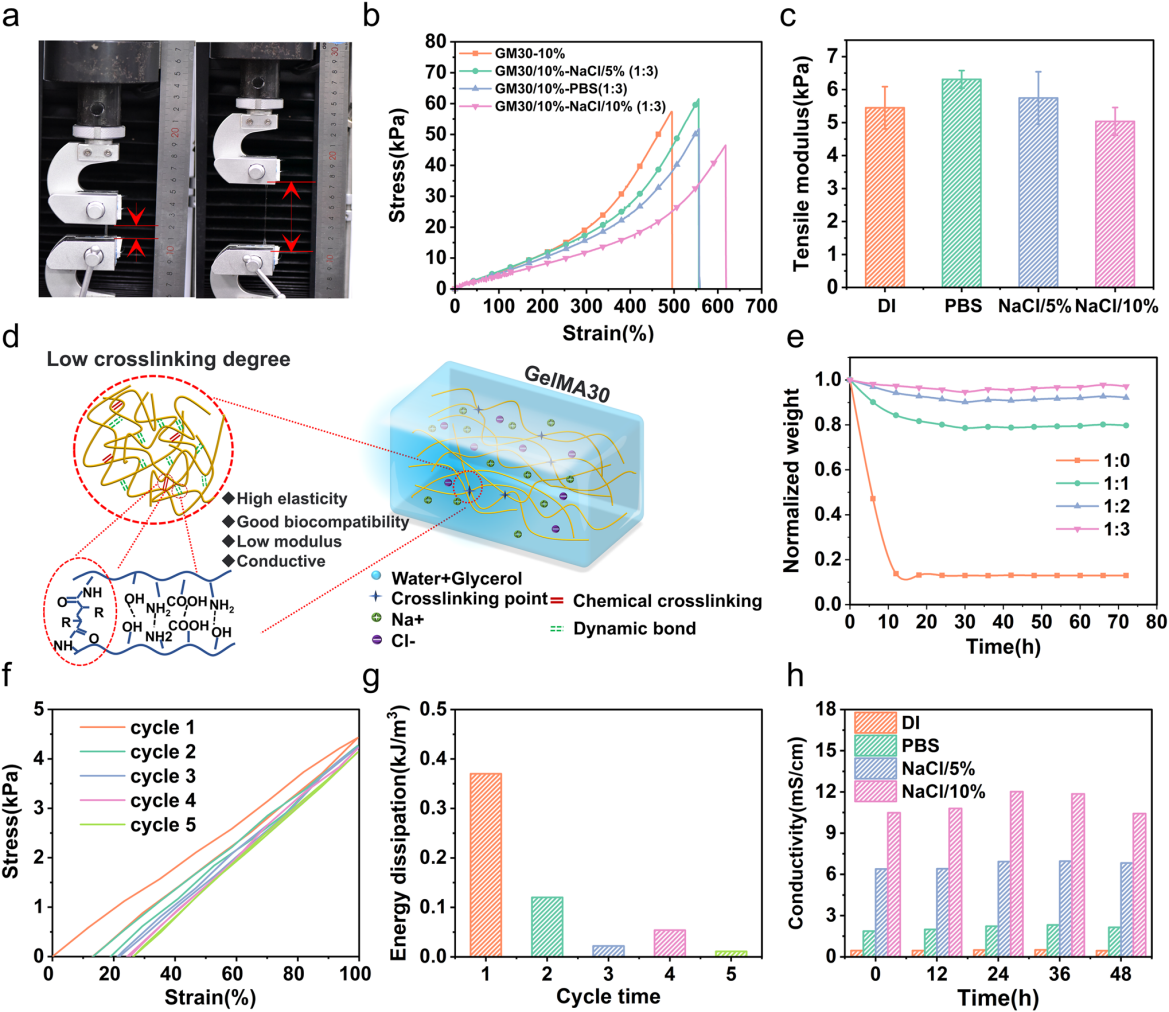
Mechanical properties of GelMA30 (10% w/v) hydrogel**. a** Stretching image of GelMA30. **b** and **c** Stress–strain curves of GelMA30 (W: G = 1: 3) with different ion concentrations and the corresponding tensile modulus. **d** The interior structure of GelMA30. **e** The relative weight loss of GelMA30 under different glycerol ratios. **f** The continuous cyclic loading curve of GelMA30 (W: G = 1: 3) at 100% strain. **g** Corresponding energy dissipation of the GelMA30 (W: G = 1: 3) at 5 cycles loading 100% strain. **h** GelMA30 (W: G = 1: 3) conductivity with different ion concentrations at each time point

In this study, GelMA30 hydrogel was developed by altering the amino group ratio on the molecular chain of gelatin, and the ratio of the amino group replaced by methacryloyl was 30% to create an ultra-low modulus of biohydrogel matrix. GelMA30 at a concentration of 10% exhibits favorable characteristics such as elasticity, biocompatibility, and a low modulus [40]. GelMA30 (10% w/v) was dissolved in water and glycerol binary solvent containing phenyl-2, 4, 6-trimethyl benzoyl phosphite lithium (0.3% w/v). The hydrogel's interior structure is shown in Fig. 2d. The GelMA30 molecular chains were long and closely entangled. Due to the low degree of crosslinking, more free space and many reversible dynamic bonds exist, such as hydrogen bonds and amino groups between molecular chains. Fewer covalent chemical crosslinking linkages maintain an entire network. At the same time, the dissociation and rebuilding of reversible dynamic bonds offer an effective means of energy dissipation when GelMA30 hydrogel is exposed to strain. Therefore, GelMA30 hydrogel with ultra-low modulus and excellent elasticity can elongate by approximately 600% (Fig. 2a, b). The free water in the hydrogel evaporates fast at room temperature. This study investigated that different glycerol ratios in binary solvents affected the water retention of the hydrogel at room temperature to avoid dehydration that could affect the mechanical properties (Fig. S1**a**). Figure 2e shows the effect of the water and glycerol (W: G) ratio on the GelMA30 relative weight loss (RWL). As the ratio decreased, the RWL of GelMA30 decreased. The results showed that the RWL of GelMA30 decreased from 87.04% (W: G = 1: 0) to 2.89% (W: G = 1: 3) after storing for 72 h at room temperature. After 6 h, GelMA30 (W: G = 1: 3) RWL showed no change. As shown in Fig. S1**b**, the GelMA30 ionic hydrogel was transparent, allowing aesthetic visualization and inspection of electronic devices.

Conductivity is an essential metric for wearable and implanted sensors. In Fig. 2h, when Phosphate Buffer Saline (PBS) was added to GelMA30, the conductivity of GelMA30 was 1.87 mS cm^−1^. PBS can be swapped out for NaCl to increase ionic conductivity. The elastic modulus of GelMA30 showed no appreciable change when the NaCl content rose from 5% to 10%, but the conductivity increased from 6.39 to 10.48 mS cm^−1^ at 0 h. The findings also demonstrated that the conductivity of hydrogel was nearly constant from 0 to 48 h, indicating that the hydrogel may be used as a sensor for long time monitoring. Because the low crosslinking density provides a channel for ion migration, the GelMA30 (10% NaCl) ionic conductivity is higher than that in the published literature. For example, Han et al. [41] designed and synthesized the PAM/PBA-IL/CNF ionic conductive hydrogel (6.94 ± 0.21 mS cm^−1^). In Fig. 2c, GelMA30 (W: G = 1: 3, 10% NaCl) exhibited an ultra-low tensile modulus of 5.03 ± 0.42 kPa.

The elasticity of hydrogel was characterized by cyclic tensile loading and unloading 100% strain experiments. Figure 2f shows a clear hysteresis loop without fracture. Due to the remodeling of dynamic bonds, GelMA30 can be restored. As shown in Fig. 2g, the maximum energy dissipation in the first stretching cycle was 0.37 kJ m^−3^. In comparison, the energy consumption in the second cycle was 0.12 kJ m^−3^. At the fifth time, due to stable covalent bonding and remodeling of dynamic bonds, the energy dissipation decreased by approximately 0.01 kJ m^−3^. The weaker dynamic bonds in the internal molecular chain network were destroyed under the first and second stretching, causing more significant energy dissipation. GelMA30 (W: G = 1:3) demonstrated ultra-low modulus and high elasticity, which is suitable for the matrix of composite materials. The ionic conductivity and dehydration performance are essential properties of flexible strain sensors. Therefore, the GelMA30 hydrogel (10%, W: G = 1:3) was used in the study as an ultra-low modulus matrix to be embedded by UFNs.

### Adhesion and Biocompatibility of GelMA30 Hydrogel

**Fig. 3:**
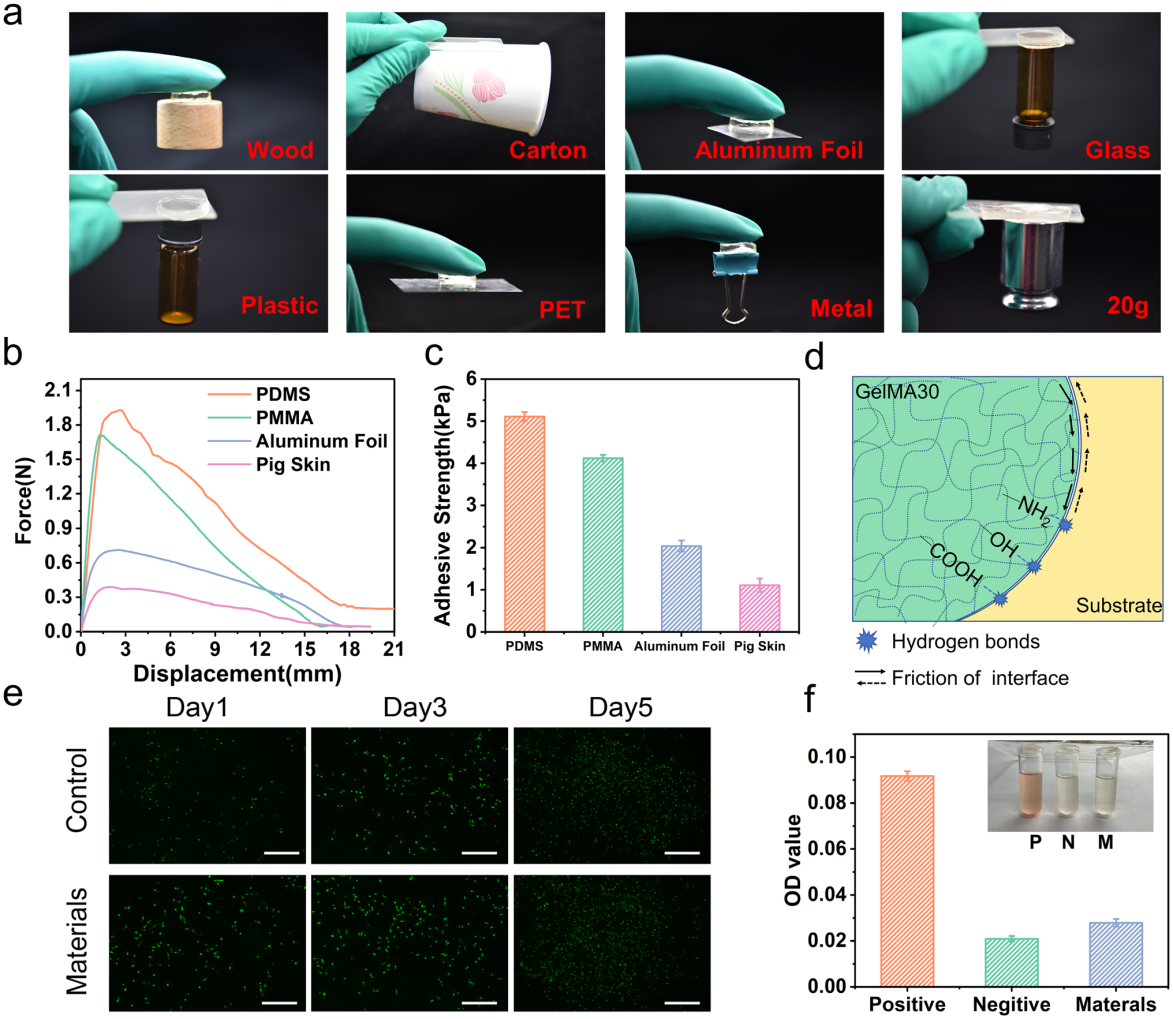
Adhesive properties of the GelMA30 hydrogel.** a** Demonstration of GelMA30 hydrogel adhering to different substrates. **b, c** The force–displacement curves of GelMA30 hydrogel adhered to four substrates and corresponding adhesive strengths. **d** Schematics of adhesive mechanism. **e** Live/dead fluorescence images of L929 cells at different times. **f** Hemolytic evaluation of GelMA30 hydrogel and the representative images of the hemolysis test

GelMA30 hydrogel exhibits excellent mechanical properties and adheres to the surface of various materials, such as wood, paper, aluminum, glass, and plastic (Fig. 3a). The highest adhesive forces of the hydrogel to the four substrates (Polydimethylsiloxane (PDMS), Polymethylmethacrylate (PMMA), aluminum foil, and pig skin) were 1.92, 1.54, 0.77, and 0.42 N, respectively (Fig. 3b). The maximum adhesive strength to PDMS was 5.11 ± 0.11 kPa (Fig. 3c). Due to the abundant activated reversible dynamic bonds (such as amino and hydroxyl) on the surface of the hydrogel and friction force between the hydrogel and substrate (Fig. 3d), the hydrogel could adhere to different materials.

Since the biocompatibility and blood compatibility of hydrogel is critical as an implantable bioelectronic, the fibroblast cell (L929) proliferation value was used to validate the biocompatibility and toxicity of GelMA30 hydrogel. In Fig. 3e, two experimental groups (Blank and Material group) were set up to study the live and dead fluorescence images of cells cultivated for 1, 3, and 5 days. The number of cells increased significantly as culture time continued, and almost no dead cells existed. The OD value of the material group increased substantially with time, demonstrating that the hydrogel exhibited good biocompatibility (Fig. S1**c**). Next, rabbit red blood cells were used to test the hemolysis of hydrogel. In Fig. 3f, the red solution appeared in the positive control group. The supernatant of the material group was almost colorless, and the OD value was close to the negative control group. Therefore, the GelMA30 meets the requirements of safe materials. The results showed many active groups on the surface of GelMA30, which was suitable for fixing the conductive circuit by a covalent bond. The adhesive characteristic of GelMA30 hydrogel provided an interfacial force between the fiber surface and hydrogel matrix in the composite material to synergistically enhance the strength of hydrogel.

### Adjustable Poisson's Ratio and Elastic Modulus of Gelatin-Based Film for High Conformality

GelMA30 hydrogel exhibits good biocompatibility and elasticity, but it is challenging to fabricate film due to its poor toughness (Movie S1). Two critical issues must be addressed to ensure high conformality and tight attachment between biohydrogel electronic and tissues: (1) Decreasing the hydrogel's thickness while maintaining the film's high toughness; (2) Endowing GelMA30 films with NPR effect. Therefore, the measure that the UFNs were embedded into the ultra-low modulus matrix was taken to address the above issues. First, by varying the UFN volume fraction in the hydrogel, the hydrogel's elastic modulus flexibly matched the elastic modulus of soft tissues. Meanwhile, embedding UFNs improved hydrogel's toughness in fabricating a film. Subsequently, altering the UFNs' topological structures endowed hydrogel with metamaterial properties and a J-shaped stress–strain response.

**Fig. 4:**
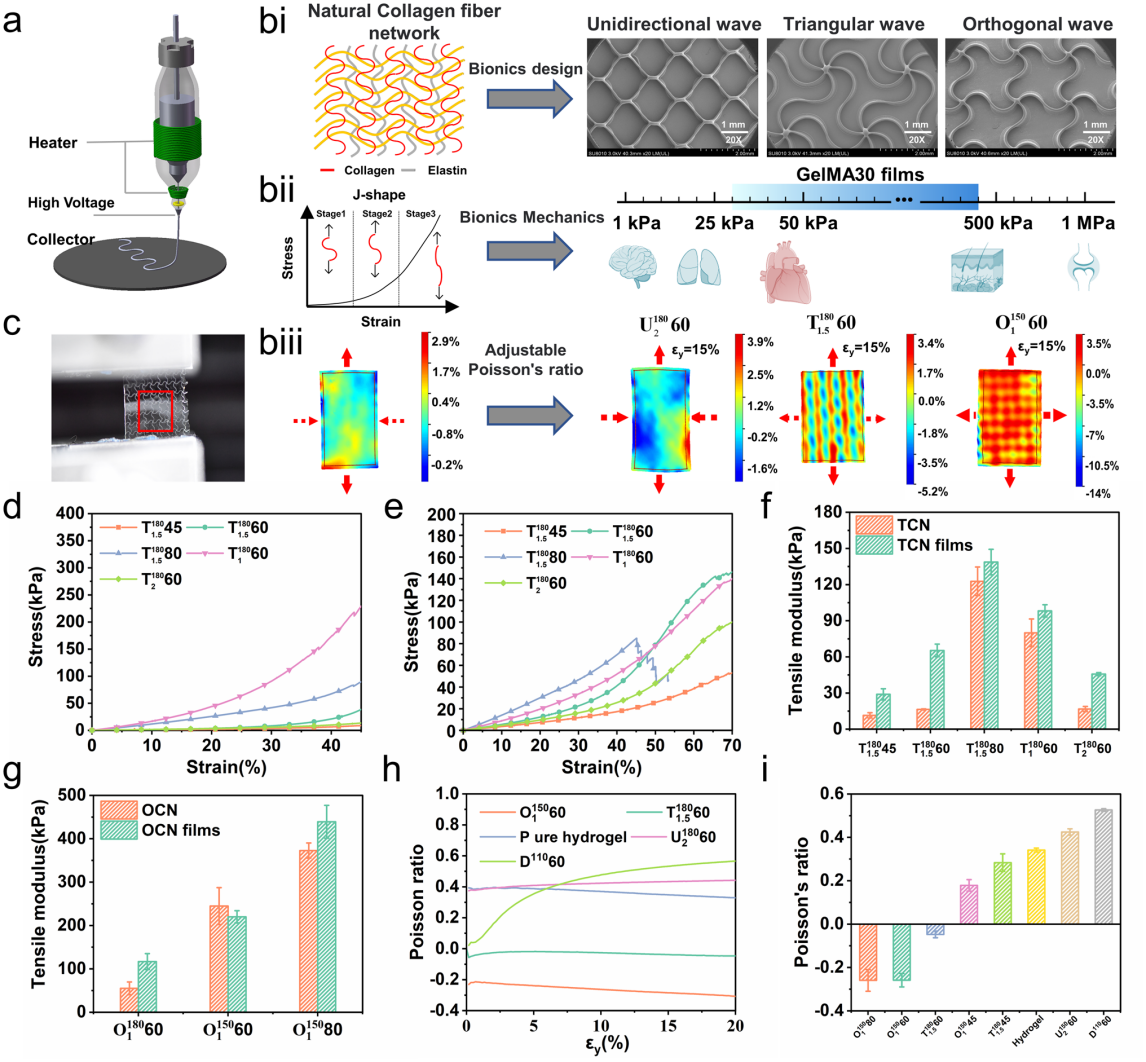
Adjustable mechanical properties of GelMA30 films. **a** Schematic of MEW. **bi** Bionic design pattern (unidirectional, triangular, and orthogonal waves). **bii** Adjustable elastic modulus range of GelMA30 films. **biii** The strain cloud of the pure hydrogel, ${\text{U}}_{2}^{{{180}}} {60}$, ${\text{T}}_{1.5}^{{{180}}} {80}$ and ${\text{O}}_{1}^{{{150}}} {60}$ films. **c** Stretching image of the OCN film. **d, e** Stress–strain curves of the TCN and TCN films. **f** Elastic modulus of the TCN and TCN films. **g** Elastic modulus of the OCN and OCN films. **h** The relationship between the Poisson's ratio of different UFN films with longitudinal strain. **i** The Poisson's ratio of different UFN films at 15% strain

Many biological tissues contain a collagen fiber network structure with bending and chain properties (Fig. 4bi). Three types of UFNs were designed considering the wavy shape of collagen fibers with unidirectional (U), triangular (T), and orthogonal (O) waves. UFNs were printed by MEW at critical speeds (Fig. 4a). UFNs consist of a basic geometry unit that has two identical arcs or sine waves with an arc angle of θ degree, arc diameter of D (mm), and fiber diameter of W (µm) (Fig. S2**a**). The expansion direction of the wave is elastic (Fig. S2**bi**). The symbol ${\text{U}}_{{\text{D}}}^{{\uptheta }} {\text{W}}$ is the unidirectional corrugated networks, where D (mm) is the arc diameter, θ is the arc angle, and W (µm) is the fiber diameter. Three types of PCL stretchable UFNs were subjected to uniaxial loading at 40% strain in the elastic direction. The triangular and orthogonal corrugated networks (TCN and OCN) demonstrated NPR effect (Figs. **S2e** and **S3ai**). Hence, the film embedded with TCN or OCN can exhibit NPR effect, demonstrating high conformality with soft tissues. As the strain increased, the unidirectional corrugated networks (UCN) contracted axially, showing a positive Poisson's ratio effect (Fig. S2**c**). GelMA30 hydrogel performed high viscosity due to glycerol, and the UFNs were embedded in the hydrogel matrix by layer-by-layer scraping to fabricate film with a thickness of 500 µm (Movie S2). The GelMA30 films with UFNs demonstrated high toughness (Movie S3).

A non-contact 2D strain measurement method estimated Poisson's ratio of the film. In Fig. 4c, when the films were subjected to longitudinal strain, the films' strain cloud in the red frame was reconstructed, and the Poisson's ratio was calculated by Digital Image Correlation (DIC) software. The DIC software reconstructed the strain cloud when the pure hydrogel was subjected to 15% longitudinal strain. In Fig. 4biii, the pure hydrogel showed axial contraction, and the majority area of the strain cloud performed negative transverse strain. The average Lagrange axial strain (E_XX_) was negative and linearly declined as the longitudinal strain (ε_y_) increased, as shown in Fig. S3**e**. Figure 4i shows the positive Poisson's ratio of pure hydrogel (0.341 ± 0.009). The topological structure was changed to increase the axial contraction of the film to simulate the large Poisson's ratio (> 0.5) of the tendon or ligament. This study printed a 110-degree diamond network (Figs. S2**ai** and **biv**). The Poisson's ratio of its composite film was 0.526 ± 0.005 (Fig. 4i).

Because of the corrugated units in UFNs and the ultra-low modulus of GelMA30 hydrogel, the UFN fflms showed a J-shaped stress–strain response like the actual tissues. As shown in Fig. 4bii, the stress changed in three stages when the films were loaded in the elastic direction. In the first stage, the deformation generated by the expansion of the corrugated fibers produced a low elastic modulus region. The fibers' spin and twist occurred in the second stage, resulting in more significant stress. In the third stage, the stress increased dramatically because of the fibers' deformation. Due to the plastic material of PCL, the third stage was the failure stage.

To regulate the mechanical properties of GelMA30 films flexibly, further investigation of the mechanism of stretchable UFNs to enhance hydrogel strength is necessary. Since the interface force between the surface of fiber and hydrogel significantly impacts the hydrogel strength. Therefore, the composite fiber diameter influences the UFN volume fraction and strength. In Fig. 4d, three experimental groups of TCNs are shown with different fiber diameters (45, 60, and 80 µm). When TCNs experienced strain in the elastic direction, the stress–strain curve of pure networks demonstrates a typical J-shaped response. The ${\text{T}}_{1.5}^{{{180}}} {45}$ elastic modulus was less than 20 kPa within 40% strain, while the ${\text{T}}_{1.5}^{{{180}}} {60}$ elastic modulus was approximately 20 kPa under 10% strain. The results reveal that as the fiber diameter increases, the flexibility of the UFNs decreases. When the TCN and UCN films experienced uniaxial tensile strain, Figs. 4f and **S3d** showed that the films' elastic modulus increased with the fiber diameter or arc diameter increasing, and the maximum elastic modulus of the ${\text{T}}_{1.5}^{{{180}}} {80}$ film was 138.68 ± 10.61 kPa. Figure 4e shows that the strain of experimental groups reaches around 45% without breaking, thus achieving the maximum strain of most soft tissues (for example, the ultimate strain of skin is 30%).

The Poisson's ratio of the films was investigated with the different topological structures of the UFNs. The hydrogel's strain was redistributed when TCN and OCN with NPR effects were embedded into a hydrogel matrix. In Fig. 4biii, the strain cloud showed that the majority area of the ${\text{O}}_{1}^{{{150}}} {60}$ and ${\text{T}}_{1.5}^{{{180}}} {60}$ films was a positive strain. The average Lagrange strain E_YY_ or E_XX_ and ε_y_ curves are linear, with values greater than 0 (Fig. S3**g, h**). As shown in Fig. S3**aii**, the ${\text{O}}_{1}^{{{150}}} {60}$ film expands axially. In Fig. 4i, the Poisson's ratio of the ${\text{T}}_{1.5}^{{{180}}} {60}$ film (-0.047 ± 0.015) was greater than the ${\text{O}}_{1}^{{{150}}} {60}$ film (− 0.25 ± 0.031); Therefore, flexible sensors with NPR substrate avoid delamination and prevent failure interface between tissue and flexible sensors and improve sensors' sensitivity. Kim et al. [42] embedded an expandable structure consisting of glass fiber into the elastic matrix, causing the elastic matrix to have NPR effect. They developed a flexible substrate analogous to the skin, solving the delamination problem induced by human skin deformation. Liu et al. [43] fabricated the piezoresistive sensor of anisotropic porous foam with NPR effect by bidirectional freezing manufacturing technology, which improved the piezoresistive sensors' low-pressure sensitivity and signal reliability. However, it is unsuitable as an implantable flexible electronic device due to its poor biocompatibility, non-degradability and mechanical mismatch with soft tissues. In the present study, the mechanics of the ${\text{T}}_{1.5}^{{{180}}} {60}$ film with NPR effect are similar to those of the heart. The elastic modulus of the ${\text{T}}_{1.5}^{{{180}}} {60}$ film (65.3 ± 5.23 kPa) was close to that of the pericardium, and it achieves a typical J-shaped stress–strain response curve (Fig. 4e, f). As shown in Fig. 4h, the different types of films showed a steady Poisson's ratio within 15% strain. The results show that embedding UFNs with NPR effects can regulate the Poisson's ratio of hydrogels to be negative.

Furthermore, the results showed that the elastic modulus of the TCN films was higher than that of pure TCNs. For example, the elastic modulus of ${\text{T}}_{1}^{{{180}}} {60}$ (79.99 ± 11.42 kPa) was lower than that of its composite film (98.16 ± 5.11 kPa). When the TCN films experienced longitudinal strain, the TCN axial expansion resisted the hydrogel's axial contraction. In Fig. 4f, the elastic modulus of ${\text{T}}_{1.5}^{{{180}}} {45}$ (11.46 ± 2.21 kPa) was less than that of ${\text{T}}_{1.5}^{{{180}}} {60}$ (16.39 ± 0.46 kPa). As shown in Fig. S2**f**, the ${\text{T}}_{1.5}^{{{180}}} {45}$ film contracted axially. Therefore, the Poisson's ratio of the ${\text{T}}_{1.5}^{{{180}}} {45}$ film (0.28 ± 0.04) was larger than that of the ${\text{T}}_{1.5}^{{{180}}} {60}$ film (-0.047 ± 0.015) but lower than that of the pure hydrogel. The results indicate that when the elastic modulus of the stretchable UFN is larger than a critical value, the UFN axial expansion overcomes the hydrogel contraction and produces the NPR effect in a composite film. The impact of UFNs with NPR effect on the composite films' elastic modulus was explored. The Poisson's ratio of the ${\text{O}}_{1}^{{{150}}} {80}$ film was less than other films. Figure 4g shows that the elastic modulus of the ${\text{O}}_{1}^{{{150}}} {80}$ film (439.08 ± 37.82 kPa) is larger than that of other films, approximately 80 times that of the hydrogel (5.03 ± 0.42 kPa). Hence, the elastic modulus of different films was compared under the same UFN volume fraction to explore the influences of topological structures on the enhancement effect of the hydrogel strength. In Table S1–**S3**, the volume fraction of ${\text{T}}_{2}^{{{180}}} {60}$ (4.30%) was approximately equal to that of ${\text{U}}_{{2}}^{{{180}}} {60}$ (4.23%) under the same fiber diameter of 60 µm, and the elastic modulus of ${\text{T}}_{2}^{{{180}}} {60}$ (16.70 ± 2.14 kPa) was smaller than that of ${\text{U}}_{{2}}^{{{180}}} {60}$ (49.71 ± 9.03 kPa). However, the elastic modulus of the ${\text{T}}_{2}^{{{180}}} {60}$ film (45.69 ± 1.25 kPa) was bigger than that of the ${\text{U}}_{{2}}^{{{180}}} {60}$ film (41.50 ± 4.2 kPa). Subsequently, two types of UFNs with NPR effect were compared to research the enhancement effect. The volume fraction of ${\text{T}}_{1}^{{{180}}} {60}$ (8.2%) was more significant than that of ${\text{O}}_{1}^{{{180}}} {60}$ (4.90%) under the same fiber diameter of 60 µm. The elastic modulus of ${\text{T}}_{1}^{{{180}}} {60}$ (79.99 ± 11.42 kPa) was larger than that of ${\text{O}}_{1}^{{{180}}} {60}$(55.04 ± 14.79 kPa), whereas the elastic modulus of the ${\text{T}}_{1}^{{{180}}} {60}$ film (98.16 ± 5.11 kPa) was less than that of the ${\text{O}}_{1}^{{{180}}} {60}$ film (116.68 ± 18.41 kPa). Therefore, UFNs with NPR greatly enhanced the hydrogel strength.

Based on the experiments above, it has been demonstrated that the elastic modulus of GelMA30 films with J-shaped stress–strain response can be adjusted within the range of 20 to 420 kPa, and their Poisson's ratio can be regulated from -0.25 to 0.52, flexibly matching the mechanics of most soft tissues. Furthermore, GelMA30 films embedded with different UFNs can be used in different applications. For example, the unidirectional corrugated network (UCN) imparts elasticity primarily in one direction, making the UCN films ideal for monitoring unidirectional motion, such as in human joints. The triangular corrugated network (TCN) retains a negative Poisson's ratio effect even with a low elastic modulus. Hence, The TCN films are suitable for matching the mechanics of ultra-soft tissues (such as the heart and lung) and can be conformal with ultra-soft tissues. In comparison, the orthogonal corrugated network (OCN) exhibits a higher elastic modulus and a more pronounced negative Poisson's ratio effect compared to other networks. The OCN films are well-suited for monitoring soft tissues with a larger elastic modulus. For instance, the ${\text{O}}_{1}^{{{150}}} {80}$ film exhibits an elastic modulus similar to that of the skin.

### Sensor Performance of Gelatin-Based Metamaterial Films

**Fig. 5:**
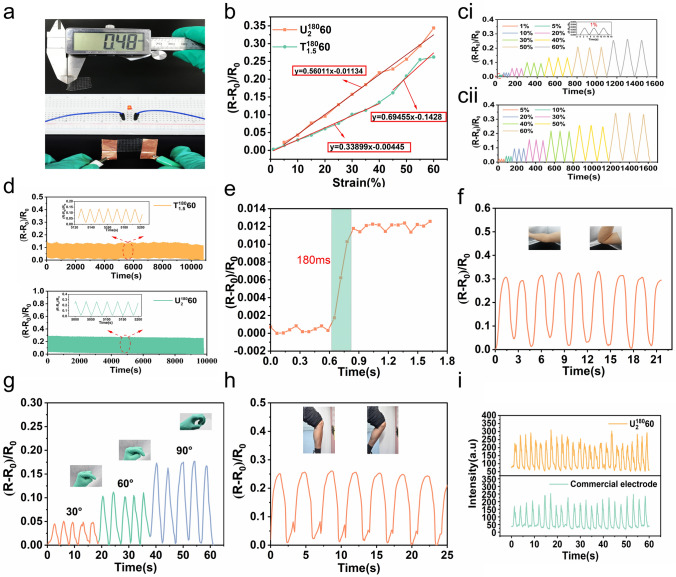
Electrical properties of GelMA30 metamaterial films and their applications as strain sensors in vitro.** a** Image of GelMA30 film connected to the LED circuit and its thickness.** b** The RCR-strain curves of the ${\text{T}}_{1.5}^{{{180}}} {60}$ and ${\text{U}}_{{2}}^{{{180}}} {60}$ films within 60% strain. **ci** and **cii** The RCR of the ${\text{T}}_{1.5}^{{{180}}} {60}$ and ${\text{U}}_{{2}}^{{{180}}} {60}$ films under different strains. **d** The RCR curves of the ${\text{T}}_{1.5}^{{{180}}} {60}$ and ${\text{U}}_{{2}}^{{{180}}} {60}$ films at 500 times cyclic loading and unloading 40% strain. **e** The response time of the ${\text{T}}_{1.5}^{{{180}}} {60}$ film. **f** The RCR curves of the ${\text{U}}_{{2}}^{{{180}}} {60}$ film adhered to the elbow **g** finger **h** and knee. **i** Muscle electrical signals measured by the ${\text{T}}_{1.5}^{{{180}}} {60}$ film and commercial electrodes

GelMA30 films perform with mechanically adjustable characteristics flexibly matching soft tissue mechanics, such as the heart. The films maintain high conformality with the ultra-soft tissue due to their metamaterial features, ensuring the effectiveness of the interface without delamination. To verify high conformality between GelMA30 film and soft tissues, The ${\text{T}}_{1.5}^{{{180}}} {60}$ metamaterial film with a J-shaped stress–strain response was used as an implantable flexible strain sensor to monitor cardiac deformation. The ${\text{U}}_{{2}}^{{{180}}} {60}$ film with unidirectional elastic deformation and anisotropy was selected to monitor the joint's motion (Fig. S3**c**). Because of the films' ionic conductivity, when the conductive film experienced strain, the film's resistance changed. In Fig. 5a, the film (thickness: 500 µm) was connected to an LED circuit, which could be illuminated to show the film's conductivity visually.

To determine sensitivity, the effectiveness of the two films as strain sensors was evaluated by estimating the resistance change rate (RCR) after exposure to various strains. For the ${\text{T}}_{1.5}^{{{180}}} {60}$ film, when the strain increased from 1% to 60%, the RCR increased from 0.27% to 26.21%, indicating that even a slight strain (1%) can be identified (Fig. 5ci). Therefore, it can measure minimal deformation. For the ${\text{U}}_{{2}}^{{{180}}} {60}$ film, as the strain raised from 5% to 60% in the elastic direction, the RCR increased from 2.25% to 34.37% (Fig. 5cii). In Fig. 5b, the ${\text{U}}_{{2}}^{{{180}}} {60}$ film strain sensitivity was 0.56. Meanwhile, the ${\text{T}}_{1.5}^{{{180}}} {60}$ film sensitivity was 0.33 within 40% strain, and the sensitivity was 0.69 beyond 40% strain. The RCR of the ${\text{U}}_{{2}}^{{{180}}} {60}$ film was considerably higher than that of the ${\text{T}}_{1.5}^{{{180}}} {60}$ film (within 40% strain), and the sensitivity of the ${\text{U}}_{{2}}^{{{180}}} {60}$ film was significantly higher than that of the ${\text{T}}_{1.5}^{{{180}}} {60}$ film. The embedded ${\text{U}}_{{2}}^{{{180}}} {60}$ increases in the axial contraction of hydrogel, but the embedded ${\text{T}}_{1.5}^{{{180}}} {60}$ extends axially and resists the axial contraction of hydrogel, thus reducing the strain of the film. Therefore, the Poisson's ratio of films impacts the strain sensitivity. Subsequently, two types of films were subjected to 500 cyclic loading and unloading within 40% strain. The RCR of films was stable, indicating that the films perform outstanding stability and durability (Fig. 5d). To monitor tissues or organs with rapid frequency of deformation, the response time of strain sensors is a critical factor. For example, adults in good health breathe 10 ~ 20 times per minute on average [44]. Heartbeats are approximately 72 times per minute at rest and 100 times during exercise. Figure 5e showed that when 2% minor strain was subjected to the ${\text{T}}_{1.5}^{{{180}}} {60}$ film at 40 mm min^−1^ speed, the response time was approximately 180 ms. The results demonstrate that the ${\text{T}}_{1.5}^{{{180}}} {60}$ film can quickly respond to high-frequency and minimal deformations, such as lungs.

The ${\text{U}}_{{2}}^{{{180}}} {60}$ film shows good strain sensor performance to monitor joint motion in vitro. In Fig. 5g, the ${\text{U}}_{{2}}^{{{180}}} {60}$ film adhered to the index finger, and as the bending angle of the index finger increased from 30° to 90°, the RCR increased from 5.05% to 17.68%. The ${\text{U}}_{{2}}^{{{180}}} {60}$ film identifies the minor joint strain in real time and detects large deformation. When the elbow and knee were rotated, the RCRs were 33.15% and 25.18% respectively (Fig. 5f, h). Due to the film's softness, it was used as an electrode to monitor the action potential of muscle. As illustrated in Fig. 5i, the strength of signals measured by films was no different from that measured by the commercial electrode. The results show that the GelMA30 film can identify minor electrical signals.

### Gelatin-based Metamaterial Film for Monitoring Cardiac Deformation

**Fig. 6:**
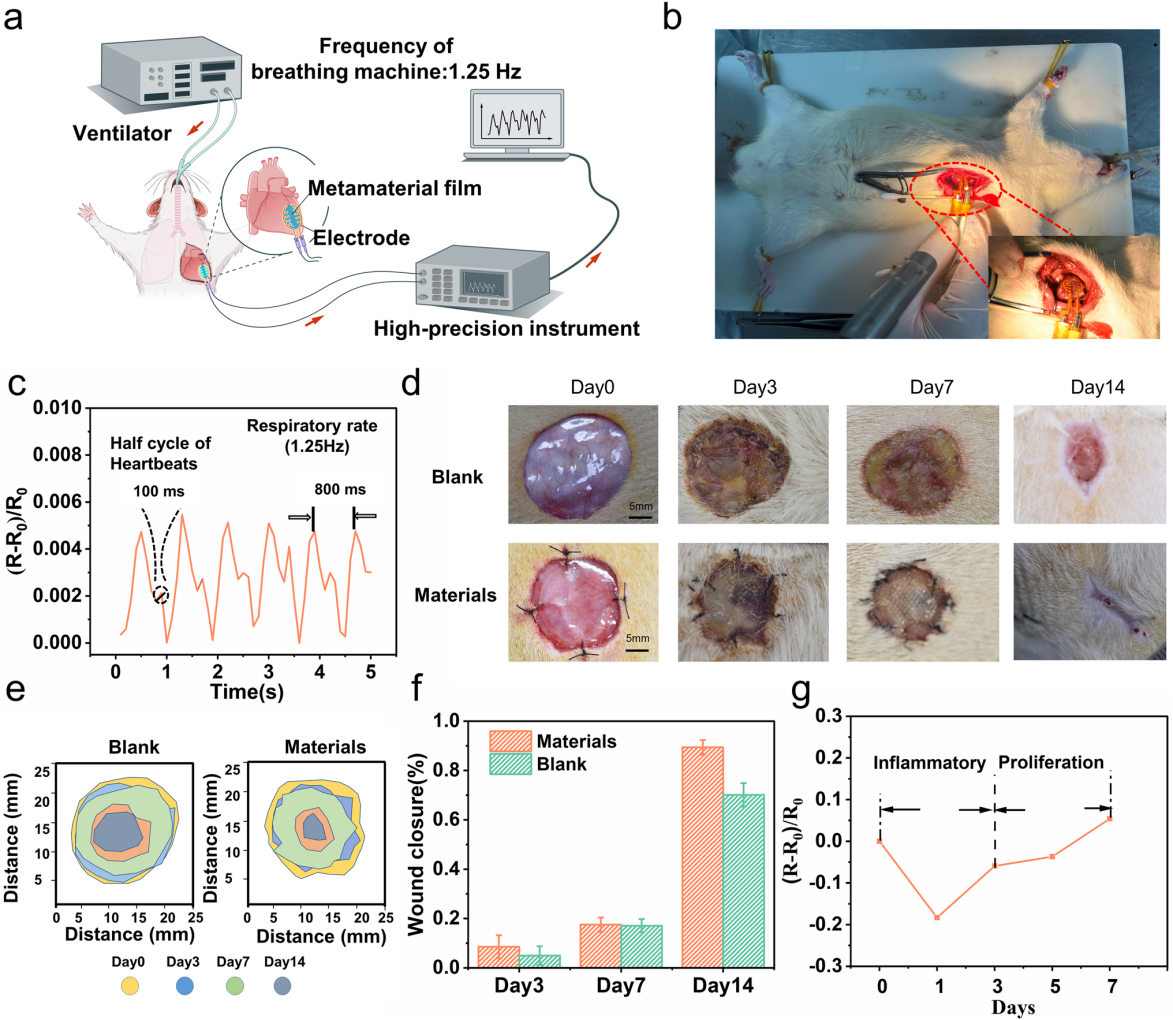
The ${\text{T}}_{1.5}^{{{180}}} {60}$ metamaterial film as a strain sensor and the repair capacity of UCN film in vivo. **a** Schematic of the ${\text{T}}_{1.5}^{{{180}}} {60}$ metamaterial film as a strain sensor to monitor cardiac deformation. **b** Image of the implanted ${\text{T}}_{1.5}^{{{180}}} {60}$ metamaterial film to monitor cardiac deformation. **c** The RCR of the ${\text{T}}_{1.5}^{{{180}}} {60}$ film induced by the cardiac deformation. **d** Representative wound images of the material and blank group on days 0, 3, 7, and 14. **e** Schematic of wound closure for each group on days 0, 3, 7, and 14. **f** The wound closure rate of the blank and material groups at each time point. **g** The RCR of the UCN film at early stage of wound healing

As shown in Fig. 6a, the ${\text{T}}_{1.5}^{{{180}}} {60}$ film and electrode were assembled into a flexible strain sensor with a diameter of 10 mm and attached to the surface of the mouse heart. In the monitoring process, a ventilator with a frequency of 1.25 Hz was used to keep anaesthetized mouse breathing. The end of the electrode was connected to a high-precision resistance instrument that monitored and recorded RCR induced by the deformation of the film in real time. The thickness of the flexible strain sensor is less than 500 µm so that the strain sensor can be tightly attached to the cardiac surface (Fig. 6b). The deformation of the mouse cardiac surface drove the ${\text{T}}_{1.5}^{{{180}}} {60}$ film deformation (Movie S4). Notably, the position of the heart and lungs is close. The expansion and contraction of the lung caused by respiration induce significant cardiac deformation. The high-precision resistance instrument recorded the RCR signals including respiration and heartbeat. Therefore, the deformation of the film included the tiny deformation induced by heartbeat and the large deformation caused by breathing. In Fig. 6c, the curve of RCR followed a periodic law, and the time interval between the peaks was 800 ms, indicating that the frequency of the curves was 1.25 Hz, consistent with the frequency of the ventilator. The findings show that the significant deformation leads to the highest value due to breathing. Because the cardiac deformation caused by breathing is more significant than the deformation caused by the heartbeat, the high-frequency heartbeat signal is not observed in the graph. Furthermore, the high-frequency heartbeat signal is superimposed into a low-frequency breath signal. Therefore, the heartbeat signal was superimposed on the maximum local value between the peaks and troughs, and the rise time for the maximum local value was half the heartbeat cycle [45]. The rise time was 100 ms, and the measured heartbeat was approximately 300 times per minute, consistent with published literature [45, 46]. The results show the tight attachment between the metamaterial film and the heart surface, ensuring the effectiveness of strain transmission. However, if the gelatin-based film is placed in the body longer and used for stable monitoring, it is essential to consider its degradation before completing the monitoring task.

### GelMA30 films for Accelerating and Monitoring Wound Healing in vivo

The experiment on full-thickness skin defect was conducted to evaluate the wound repair capability of GelMA30 hydrogel and its role in monitoring the initial stage of tissue restoration. As shown in Fig. S3**b**, the GelMA30 film can be stitched due to the embedding UFNs. As the fiber diameter increased, the suture force increased. In Figs. 6d, e, the blank and material groups wound areas steadily contracted over time, and the wound of the material group was closed on the 14th day. In Fig. 6f, the wound closure rate in the material group was 89.39%, while it was 70.11% in the blank group on the 14th day. The RCR of GelMA30 film at the wound was monitored by a high-precision resistance instrument (Fig. 6g). The RCR curve reflects the two early stages of wound healing. During the outbreak of inflammation, a large amount of tissue fluid that contains many ions infiltrates the film, increasing the conductivity of the film. After inflammation, the wound healed slowly, and the RCR of the film at the wound increased continuously. However, the main challenge faced by implantable and biodegradable hydrogel bioelectronics is the degradation of the hydrogel, which significantly impacts the accuracy of the monitoring signal. GelMA with a concentration of 10% and above typically takes more than 1–3 months to degrade in vivo completely, and it degrades less than 12% within 7 days [40]. Furthermore, early monitoring is crucial for wound monitoring to ensure that the wound is not in a constant inflammatory stage. The wound heals slowly once the inflammatory stage has passed, and continuous monitoring is unnecessary. Therefore, signal compensation is not required during the initial stage of tissue restoration. The 7-day wound signals were only collected to maintain the relative accuracy of the monitoring signal. If prolonged monitoring is needed in other applications, GelMA hydrogel with a higher crosslinking degree and concentration can be selected, or a hydrophobic coating can be applied to the surface of GelMA to enhance its stability. Additionally, appropriate signal compensation should be implemented to avoid signal errors caused by degradation.

**Fig. 7:**
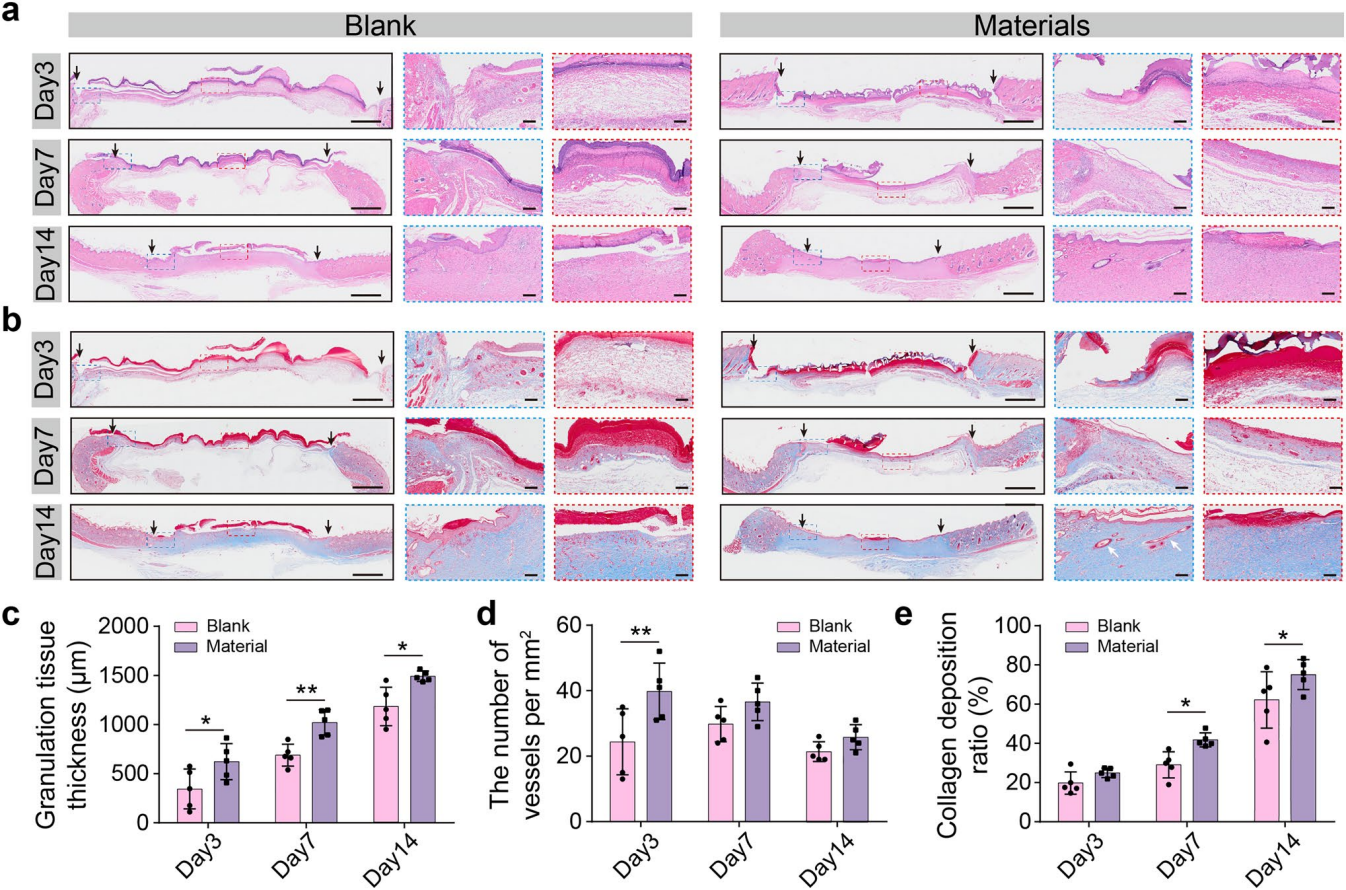
Histomorphology evaluation of wound regeneration.** a** and **b** Micrographs of PI stained with H & E and Masson on days 3, 7, and 14. The blank arrow indicates the edge of the defect area. **c** Granulation thickness of wounds. **d** The number of vessels. **e** Collagen deposition ratio of two groups. n = 5, * P < 0.05, ** P < 0.01. bar = 2 mm and 200 μm

Wound healing is a complex process supported by many different tissues and cells. In the preliminary stages of wound healing, new capillaries and fibroblasts form granulating tissue, accompanied by inflammatory cell infiltration. In Fig. 7a–c, after 3 days of treatment, histological sections of the blank group showed that the wound surface was covered with an inflammatory exudation band and numerous neutrophils at the edge, while the wound in the material group was covered with material, with fewer inflammatory cells and prominent granulation tissue. The granulating tissue thickness of the material group (622.69 ± 183.13) was significantly higher than that of the blank group (*p* < 0.05). After 7 days, the blank group showed many neutrophils in the inflammatory granulation tissue, while the material group showed more new collagen fibers at the wound site, and the granulation tissue thickness of the material group (1021.59 ± 130.57) was significantly bigger than that of the blank group (589.33 ± 111.97). On day 14, the wound surface of the material group was entirely covered by an epithelial layer, consistent with the observations in Fig. 6d, with abundant collagen content and some hair follicle structure, indicating better wound healing. Although the wound in the blank group was reduced, the wound epidermis was incomplete, and the tissue thickness was lower than that in the material group. Collagen content was quantified by calculating the area of positive blue staining. Compared with that in the blank group, the collagen content in the materials group was significantly increased (Fig. 7e). Angiogenesis is an essential parameter for wound regeneration. The number of blood vessels in the material group was higher than that in the blank group, especially on the third day (Fig. 7d). Immunohistochemical staining was used to detect the expression of CD31, a classical marker of vascular endothelial cells (Fig. S4**a, b**), which was consistent with the counting results. The material provides a better moisturizing effect while isolating the stimulation brought by the external environment, causing less inflammation. Therefore, the histomorphology evaluation of wound regeneration ensured that GelMA30 film could accelerate wound healing.

## Conclusion

In summary, the study demonstrated a mechanically programmable gelatin-based film with high biocompatibility. Ultra-low elastic modulus GelMA30 hydrogel was prepared by controlling the amino substitution rate of 30% as a matrix. The mechanics of GelMA30 hydrogel was adjusted by embedding the bionic design of UFNs. The elastic modulus was regulated from 20 to 420 kPa, and the Poisson's ratio was achieved from -0.25 to 0.52 by changing the UFN volume fraction and topological structure. The results showed that UFNs with NPR significantly affect the synergistic enhancement of hydrogel strength. A GelMA30 film with NPR and matching cardiac mechanics was designed as a strain sensor. The rats' heartbeat and respiratory rate were measured successfully to confirm the metamaterial design had high conformality between biohydrogel electronics with ultra-soft tissue and provided the effectiveness of the interface. The metamaterial biohydrogel composite system offers excellent prospects for the next generation of implantable bioelectronics to improve the signal-to-noise ratio and achieve tissue repair while monitoring.

## Supplementary Information

Below is the link to the electronic supplementary material.

Supplementary file5 (PDF 602 KB)

Supplementary file2 (MP4 39900 KB)

Supplementary file3 (MP4 226906 KB)

Supplementary file4 (MP4 30021 KB)

Supplementary file1 (MP4 18683 KB)
