# Supplementary material for: Gelatin-Based Metamaterial Hydrogel Films with High Conformality for Ultra-Soft Tissue Monitoring
Source: Nanomicro Lett. 2023 Nov 29;16:34. doi: 10.1007/s40820-023-01225-z (PMC10686972; doi:10.1007/s40820-023-01225-z)
Supplement: Supplementary file 1 — Supplementary file5 (PDF 602 KB) [file 40820_2023_1225_MOESM1_ESM.pdf]

Supporting Information for

Gelatin-Based Metamaterial Hydrogel Films with High Conformality for Ultra-Soft Tissue Monitoring

Yuewei Chen[1,2,†], Yanyan Zhou[3,†], Zihe Hu[3], Weiying Lu[3], Zhuang Li[1], Ning Gao[3], Nian Liu[1], Yuanrong Li[1], Jing He[1], Qing Gao [1], Zhijian Xie [3,*], Jiachun Li [2,*], Yong He[1,*]

[1] State Key Laboratory of Fluid Power and Mechatronic Systems, School of Mechanical Engineering, Zhejiang University, Hangzhou 310027, P. R. China

[2] School of Mechanical Engineering, Guizhou University, Guiyang 550025, P. R. China

[3] Stomatology Hospital, School of Stomatology, Zhejiang University School of Medicine, Clinical Research Center for Oral Diseases of Zhejiang Province, Key Laboratory of Oral Biomedical Research of Zhejiang Province, Cancer Center of Zhejiang University, Hangzhou 310006, P. R. China

[†]Yuewei Chen and Yanyan Zhou contributed equally to this work.

* Corresponding authors. E-mail: yongqin@zju.edu.cn (Yong He); xzj66@zju.edu.cn (Zhijian Xie); jcli@gzu.edu.cn (Jiachun Li)

Supplementary Figures and Tables

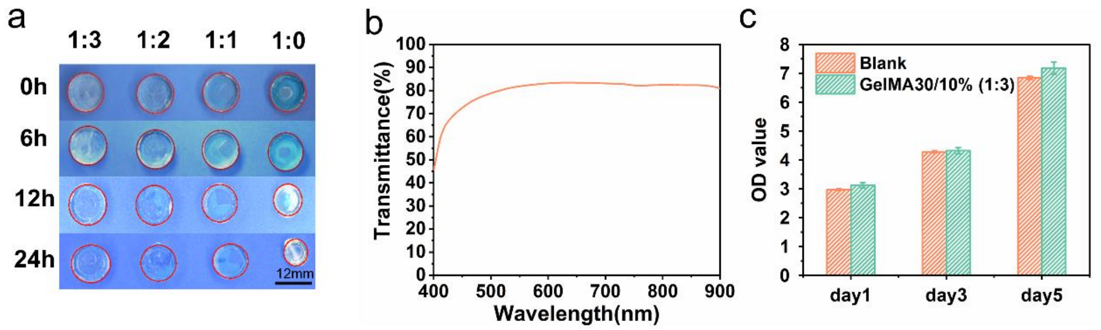

Fig. S1 Other properties of GelMA30 hydrogel. **a** Image of hydrogel dehydration with different glycerol ratios at each time point. **b** Transmittance of GelMA30 hydrogel in the visible wavelength range of 400~900 nm. **c** The absorbance at 450 nm using CCK-8

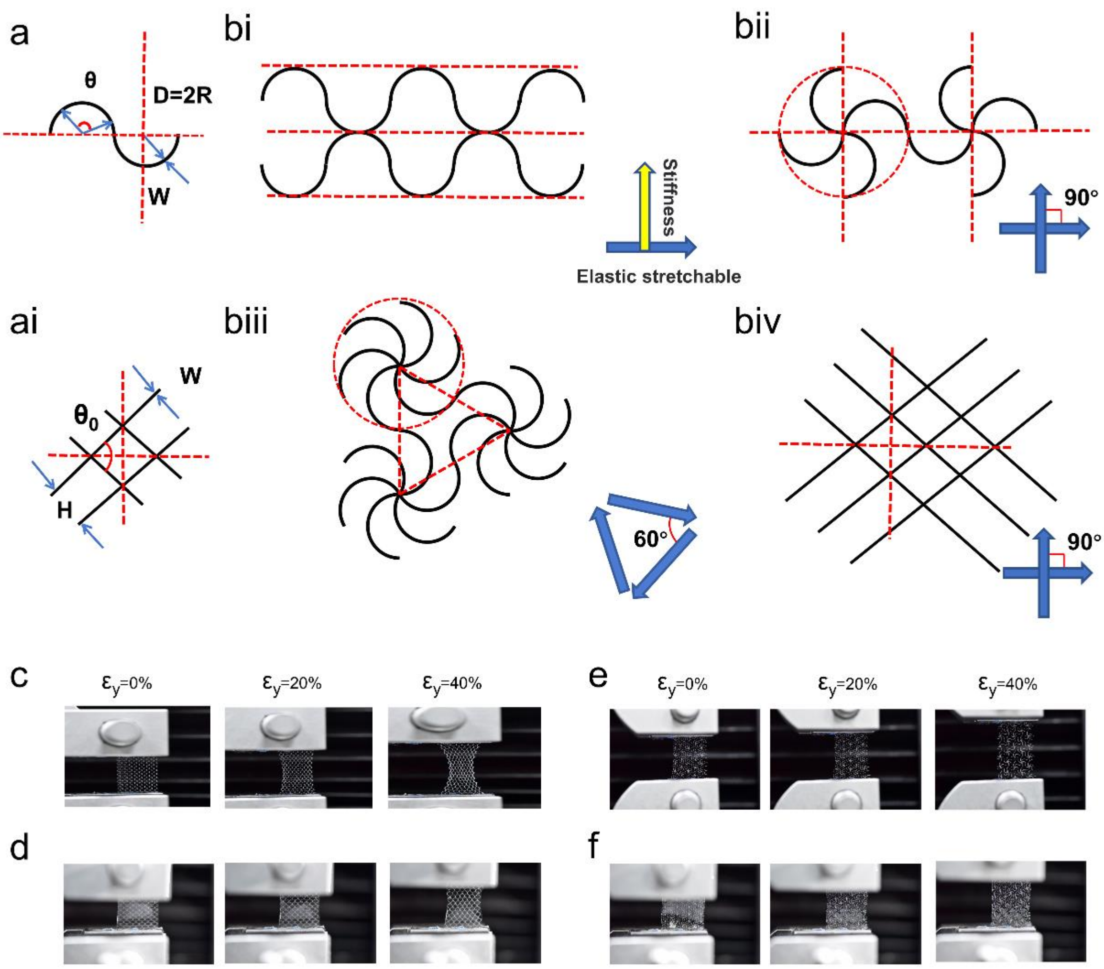

Fig. S2 Design of UFNs and images of UFNs under different tensile strains. **a** Biomimetic corrugated unit. **ai** rhombic unit. **bi** Design of unidirectional wave, **bii** orthogonal wave, **biii** triangular wave, **biv** and diamond. **c** Images of the $U_{2}^{180}60$, **d** $U_{2}^{180}60$ film, **e** $T_{1.5}^{180}45$, f) $T_{1.5}^{180}45$ film under different tensile strains

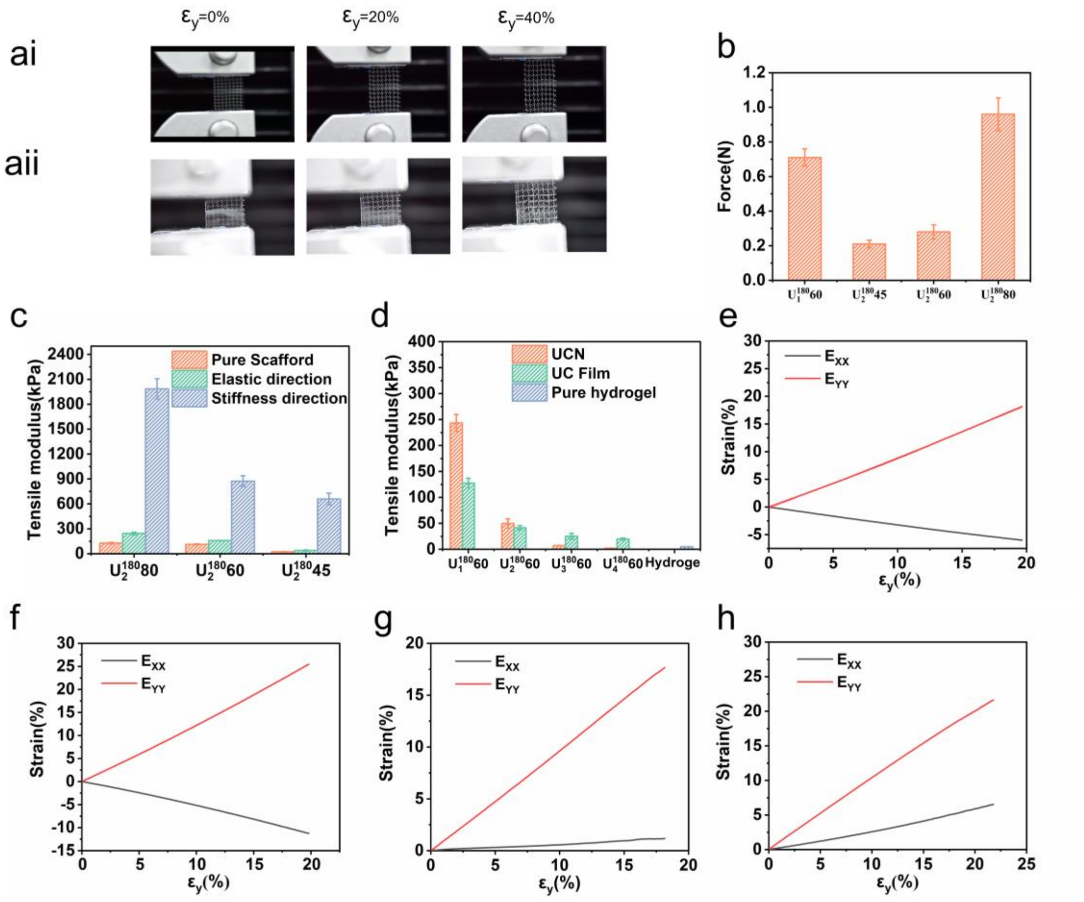

Fig. S3 Mechanical Properties of GelMA30 films and images of the $O_1^{150}60$ and $O_1^{150}60$ film under different tensile strains. **ai** and **aii** Images of $O_1^{150}60$ and its composite film under different strains. **b** The maximum suture force of the UCN films. **c** Anisotropic mechanics of the UCN films. **d** Elastic modulus of the UCN and UCN films. **e** The DIC analysis results about Lagrange average strain axial E_{XX}-ε_y and longitudinal E_{YY}-ε_y curves for the pure hydrogel, **f** $U_2^{180}60$ film, **g** $T_{1.5}^{180}80$ film, and **h** $O_1^{150}60$ film

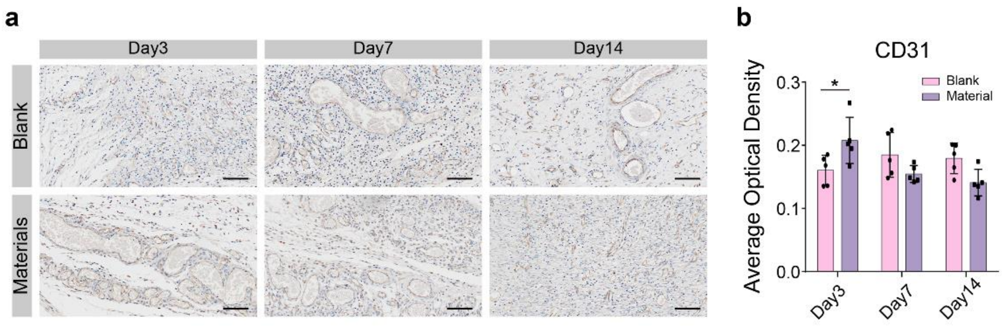

Fig. S4 CD31 staining images and semiquantitative of skin wounds. **a** Images. **b** Semiquantitative

Table S1 Volume fraction of UCN in composite films

UC films	$U_1^{180}60$	$U_2^{180}60$	$U_3^{180}60$	$U_4^{180}60$
Volume fraction (%)	**0.077**	**0.0423**	**0.028**	**0.025**

Table S2 Volume fraction of OCN in composite films

OC films	$O_1^{180}60$	$O_1^{150}60$	$O_1^{150}80$
Volume fraction (%)	**0.049**	**0.037**	**0.066**

Table S3 Volume fraction of TCN in composite films

TC films	$T_2^{180}60$	$T_{1.5}^{180}60$	$T_1^{180}60$	$T_{1.5}^{180}45$	$T_{1.5}^{180}80$
Volume fraction (%)	**0.043**	**0.055**	**0.082**	**0.038**	**0.104**

Movie S1 Fabrication of GelMA30 films without UFNs

Movie S2 Fabrication of GelMA30 films with UFNs

Movie S3 Test the toughness and elasticity of GelMA30 films with UFNs

Movie S4 Real-time monitoring of mouse cardiac deformation
